# Comparison of two related lines of tauGFP transgenic mice designed for lineage tracing

**DOI:** 10.1186/s12861-017-0149-x

**Published:** 2017-06-29

**Authors:** Linda Sharp, Thomas Pratt, Gillian E. MacKay, Margaret A. Keighren, Jean H. Flockhart, Emma J. Chandler, David J. Price, John O. Mason, John D. West

**Affiliations:** 1 Genes and Development Group, Centre for Integrative Physiology, Biomedical Sciences, University of Edinburgh Medical School, Hugh Robson Building, George Square, Edinburgh, EH8 9XD UK; 2 Genes and Development Group, Centre for Integrative Physiology, Clinical Sciences, University of Edinburgh Medical School, Hugh Robson Building, George Square, Edinburgh, EH8 9XD UK; 3 Present address: Genetics Teaching Programme, Department of Biochemistry, University of Otago, Dunedin, New Zealand; 4 Present address: Medical and Developmental Genetics Section, MRC Human Genetics Unit, MRC IGMM, University of Edinburgh, Western General Hospital, Crewe Road, Edinburgh, EH4 2XU UK

**Keywords:** Mosaic transgene expression, Green fluorescent protein, tauGFP, Adrenal cortex lineages, Preimplantation embryo, Time-lapse imaging

## Abstract

**Background:**

The tauGFP reporter fusion protein is produced nearly ubiquitously by the *TgTP6.3* transgene in TP6.3 mice and its localisation to microtubules offers some advantages over soluble GFP as a lineage marker. However, *TgTP6.3* ^*Tg/Tg*^ homozygotes are not viable and *TgTP6.3* ^*Tg/−*^ hemizygotes are smaller than wild-type. TP6.4 mice carry the *TgTP6.4* transgene, which was produced with the same construct used to generate *TgTP6.3*, so we investigated whether *TgTP6.4* had any advantages over *TgTP6.3*.

**Results:**

Although *TgTP6.4* ^*Tg/Tg*^ homozygotes died before weaning, *TgTP6.4* ^*Tg/−*^ hemizygotes were viable and fertile and only males were significantly lighter than wild-type. The *TgTP6.4* transgene produced the tauGFP fusion protein by the 2-cell stage and it was widely expressed in adults but tauGFP fluorescence was weak or absent in several tissues, including some neural tissues. The *TgTP6.4* transgene expression pattern changed over several years of breeding and mosaic transgene expression became increasingly common in all expressing tissues. This mosaicism was used to visualise clonal lineages in the adrenal cortex of *TgTP6.4* ^*Tg/−*^ hemizygotes and these were qualitatively and quantitatively comparable to lineages reported previously for other mosaic transgenic mice, X-inactivation mosaics and chimaeras. Mosaicism occurred less frequently in TP6.3 than TP6.4 mice and was only observed in the corneal epithelium and adrenal cortex.

**Conclusions:**

Mosaic expression makes the *TgTP6.4* transgene unsuitable for use as a conventional cell lineage marker but such mosaicism provides a useful system for visualising clonal lineages that arise during development or maintenance of adult tissues. Differences in the occurrence of mosaicism between related transgenic lines, such as that described for lines TP6.3 and TP6.4, might provide a useful system for investigating the mechanism of transgene silencing.

**Electronic supplementary material:**

The online version of this article (doi:10.1186/s12861-017-0149-x) contains supplementary material, which is available to authorized users.

## Background

Two lines of transgenic mice, TP6.3 and TP6.4, express CAG-tauGFP transgenes *TgTP6.3* and *TgTP6.4* respectively [1]. Both lines TP6.3 and TP6.4 were produced by electroporation of the same construct into embryonic stem (ES) cells, which were then used to generate ES cell chimaeras and founder transgenic mice. Line TP6.3 expresses a tau-green fluorescent protein (tauGFP) fusion protein almost ubiquitously. Its localisation to microtubules has some advantages over soluble GFP as a lineage marker and it is well suited for labelling axons, blood vessels and preimplantation embryos [1, 2]. This *TgTP6.3* tauGFP marker has been used to identify cell fusion between ES cells and other cell types [3] and as a lineage marker for macrophages [4], ovarian cells [5], preimplantation embryos [6] and, in particular, neural tissues [7–12].

*TgTP6.4* transgene expression was reported to be strong in many tissues yet only weak in much of the central nervous system but it was not fully characterised [1]. As *TgTP6.3* ^*Tg/Tg*^ homozygotes are lethal and *TgTP6.3* ^*Tg/−*^ hemizygotes are smaller than wild-type (WT) siblings we evaluated the second tauGFP transgenic line to determine whether *TgTP6.4* had any advantages over the *TgTP6.3* marker transgene*.* We investigated growth of *TgTP6.4* ^*Tg/−*^ hemizygotes and the viability of *TgTP6.4* ^*Tg/Tg*^ homozygotes and we characterised expression of the *TgTP6.4* transgene in embryos and adults by confocal microscopy of tauGFP fluorescence. This revealed that *TgTP6.4* ^*Tg/−*^ mice showed widespread mosaic expression. As mosaicism can be useful for analysis of clonal lineages that are established during development or by adult stem cells, we also compared mosaic expression in the adrenal cortices of *TgTP6.3* ^*Tg/−*^ and *TgTP6.4* ^*Tg/−*^ hemizygotes.

## Methods

### Mice

**Fig. 1:**
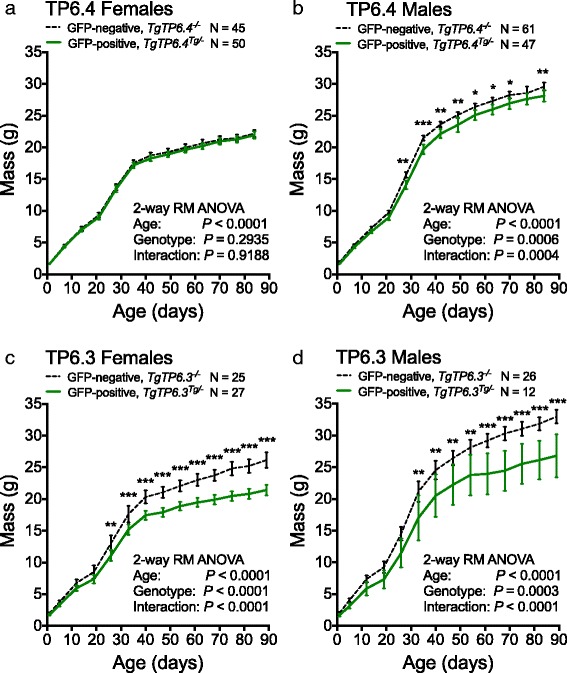
Growth of hemizygous tauGFP-positive and non-transgenic, tauGFP-negative littermates. Comparisons of postnatal body mass of hemizygous and non-transgenic (wild-type; WT) offspring from crosses between WT (C57BL × CBA/Ca)F1 females and hemizygous males: **a**, **b** GFP-positive (*TgTP6.4* ^*Tg/−*^) and GFP-negative (WT, *TgTP6.4* ^*−/−*^) females (**a**) and males (**b**). **c**, **d** GFP-positive (*TgTP6.3* ^*Tg/−*^) and GFP-negative (WT, *TgTP6.3* ^*−/−*^) females (**c**) and males (**d**). The numbers of mice are indicated above the graphs and error bars are 95% confidence intervals. Statistical significances by repeated measures 2-way ANOVA and Bonferroni multiple comparison post-tests are shown. Overall *P*-values are shown on the graphs and significant differences for comparisons between GFP-positive and GFP-negative progeny at each age are shown by asterisks: *P < 0.05; **P < 0.01; ***P < 0.001. Data for the *TgTP6.3* crosses (**c**, **d**) were published previously [2] but were re-analysed for comparison with the *TgTP6.4* crosses (**a**, **b**)

**Fig. 2:**
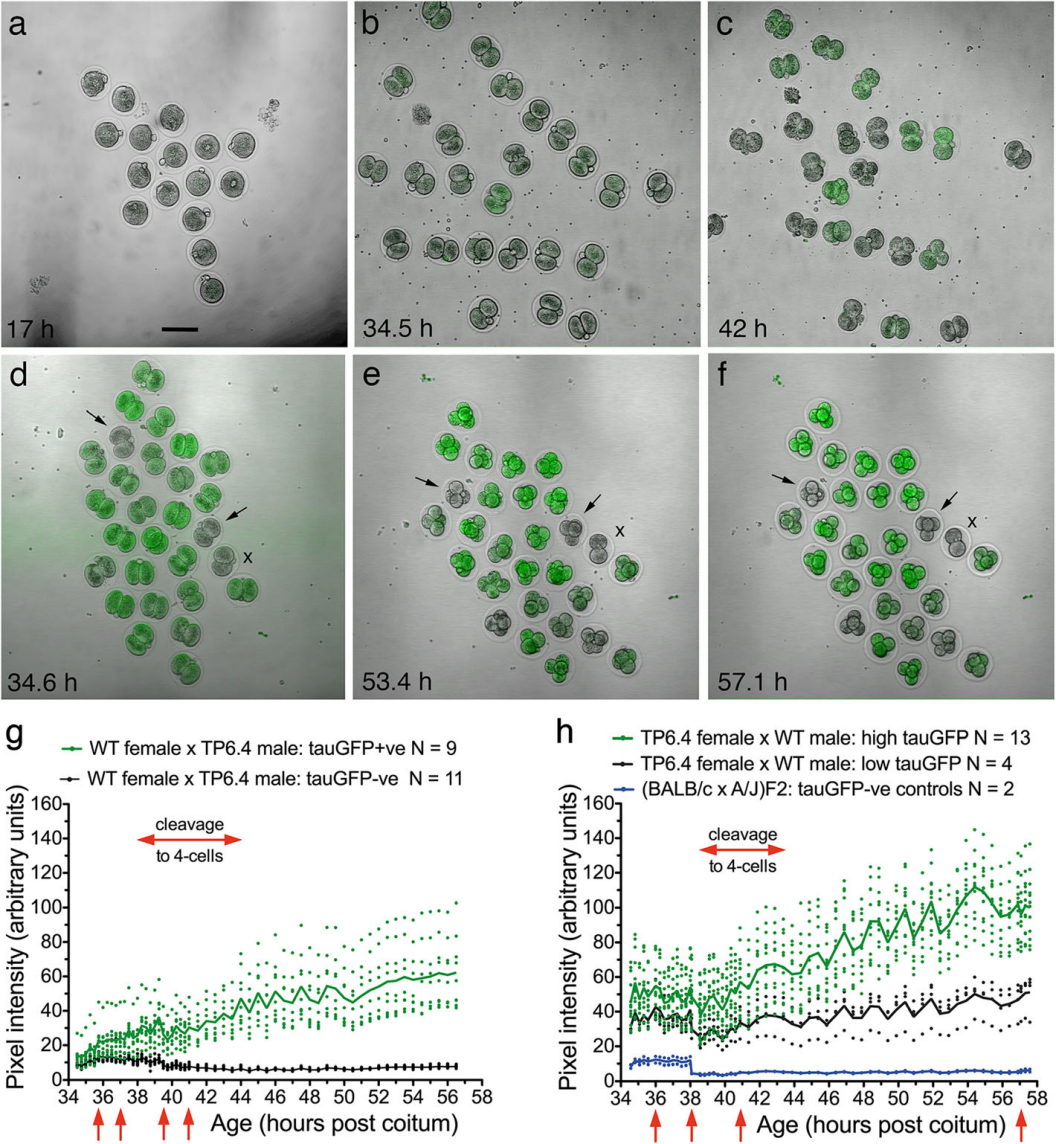
Expression of *TgTP6.4* transgene in preimplantation embryos. **a-f** Transmitted light images with overlays from the confocal FITC channel to identify tauGFP in preimplantation embryos produced by crosses between WT, (C57BL × CBA/Ca)F1 females and hemizygous *TgTP6.4* ^*Tg/−*^ males (**a-c**) and in those produced by crosses between hemizygous *TgTP6.4* ^*Tg/−*^ females and (C57BL × CBA/Ca)F1 males (**d-f**). Images shown were captured during three separate time-lapse, embryo culture experiments (shown in **a**, **b-c** and **d-f** respectively). Arrows in (**d-f**) indicate two (BALB/c × A/J)F2 negative control embryos and the embryo marked with the letter ‘x’ remained at the 2-cell stage throughout the culture period so was excluded from the quantitative study. The scale bar in (**a**) (for **a-f**) = 100 μm. **g**, **h** Plots of pixel intensity (produced by tauGFP fluorescence) at different times during embryo culture, time-lapse experiments for preimplantation embryos produced by crosses between (C57BL × CBA/Ca)F1 females and hemizygous *TgTP6.4* ^*Tg/−*^ males (**g**) and the reciprocal cross between hemizygous *TgTP6.4* ^*Tg/−*^ females and (C57BL × CBA/Ca)F1 males (**h**). The time-lapse experiments shown are representative of two such experiments for each cross. Pixel intensity values for individual embryos are shown as points. Embryos were classified as *TgTP6.4* ^*Tg/−*^ or *TgTP6.4* ^*−/−*^ according to their pixel intensity at the end of the culture period and the mean value for each genotype is shown as a solid line (upper line is *TgTP6.4* ^*Tg/−*^). In (**h**) pixel intensities are also shown for two (BALB/c × A/J)F2 negative control embryos (*lowest line* in **h**). The red arrows below the x-axis indicate times when the time-lapse was interrupted, sometimes causing differences in pixel intensity. A repeated measures 2-way ANOVA for (**g**) showed significant differences overall for age, genotype and interaction (*P* < 0.0001 in each case) and Bonferroni multiple comparison post-tests for each age showed that genotype differences were significant at 37.5 h. (*P* < 0.01), 38 h. (*P* < 0.05), and all times thereafter (*P* < 0.01 to *P* < 0.0001). For (**h**), a repeated measures 2-way ANOVA comparing embryos with high and low tauGFP levels showed significant differences overall for age, genotype and interaction (*P* < 0.0001 in each case) and Bonferroni multiple comparison post-tests for each age showed that genotype differences were significant from 42.5 h. (for 42.5–44.5 h., *P* < 0.05; for 45.0 to 47.4 h., *P* < 0.01 to *P* < 0.0001; from 47.9 h., *P* < 0.0001). A repeated measures 2-way ANOVA comparing low tauGFP and (BALB/c × A/J)F2 negative control embryos showed significant differences overall for age (*P* = 0.0093) and genotype (*P* < 0.0001) but not the interaction (*P* = 0.1831) and Bonferroni multiple comparison post-tests for each age showed that genotype differences were significant at all ages (*P* < 0.05 to *P* < 0.0001) except for 38.5, 38.6 and 39.6 h

**Fig. 3:**
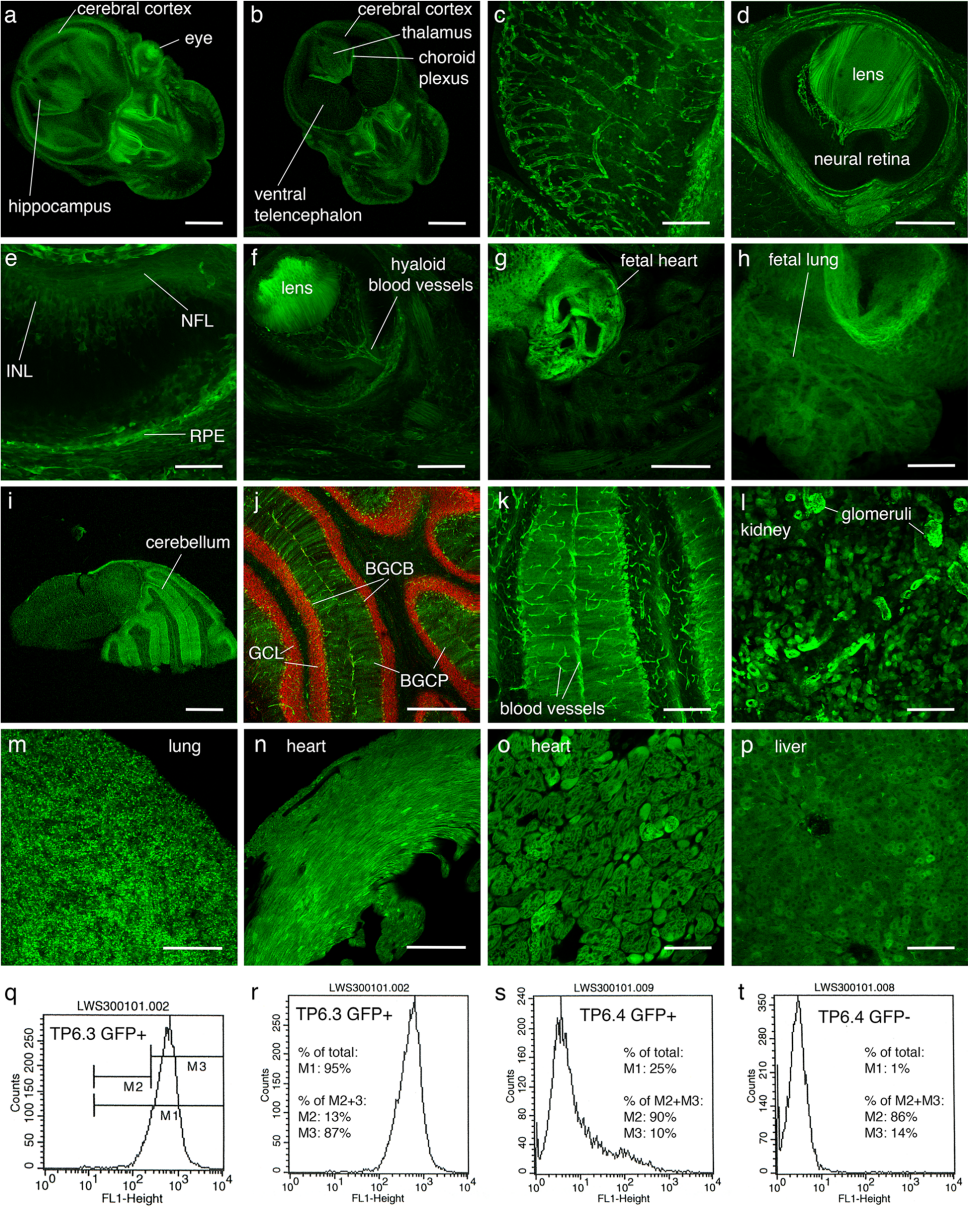
TauGFP expression in fetal and adult tissues. **a-c** Confocal images of coronal vibratome sections of heads of E13.5 tauGFP-positive fetuses showing (**a**) *TgTP6.3* ^*Tg/−*^ head, (**b**) *TgTP6.4* ^*Tg/−*^ head with reduced levels of fluorescence in the brain and (**c**) higher magnification of part of *TgTP6.4* ^*Tg/−*^ forebrain showing strong tauGFP fluorescence in blood vessels but little or none in neural tissue. **d-f** Confocal images of vibratome sections of *TgTP6.4* ^*Tg/−*^ fetal eyes, showing (**d**) tauGFP fluorescence in the lens but not the neural retina at E17.5, (**e**) higher magnification view with little or no fluorescence in most of the E17.5 neural retina but weak fluorescence in the inner nuclear layer (INL) and nerve fibre layer (NFL) and strong fluorescence in the retinal pigment epithelium (RPE) and (**f**) tauGFP fluorescence in the lens and hyaloid blood vessels but not the neural retina at E13.5. **g**, **h** Confocal images of E13.5, *TgTP6.4* ^*Tg/−*^ vibratome sections showing tauGFP fluorescence in (**g**) fetal heart and (**h**) fetal lung. **i**-**k** Confocal images of sagittal vibratome sections of adult *TgTP6.4* ^*Tg/−*^ brain showing (**i**) strong tauGFP fluorescence in the cerebellum but weaker fluorescence elsewhere, (**j**) tauGFP fluorescence and propidium iodide counterstain in the cerebellum and (**k**) higher magnification of tauGFP fluorescence in the cerebellum. **l-p** Confocal images of sagittal vibratome section of adult *TgTP6.4* ^*Tg/−*^ non-neural tissues showing tauGFP fluorescence in (**l**) kidney, with strongly-fluorescent glomeruli, (**m**) lung, (**n**, **o**) heart and (**p**) liver. **q-t** Histograms from FACS analysis of whole brains from E14.5 fetuses showing cell counts versus green fluorescence intensity (FL1-height). **q** Three gated regions defined from a *TgTP6.3* ^*Tg/−*^ tauGFP-positive sample are shown: M1 included all fluorescent cells, M2 included cells with low fluorescence (outside main fluorescent profile) and M3 included cells with high fluorescence (within main fluorescent profile). **r-t** FACS analysis showing the percentage of cells in each gated region for (**r**) tauGFP-positive *TgTP6.3* ^*Tg/−*^, (**s**) tauGFP-positive *TgTP6.4* ^*Tg/−*^ and (**t**) tauGFP-negative *TgTP6.4* ^*−/−*^, fetal brains. Abbreviations: BGCB, Bergmann glia cell body; BGCP, Bergmann glia cell processes; GCL, granule cell layer; INL, inner nuclear layer of neural retina; NFL, nerve fibre layer; RPE, retinal pigment epithelium. Scale bars: **e**, **o** = 50 μm; **p** = 100 μm; **c**, **f**, **h**, **k**, **l** = 200 μm; **d**, **g**, **j**, **m**, **n** = 500 μm; **a**, **b**, **i** = 1000 μm

**Fig. 4:**
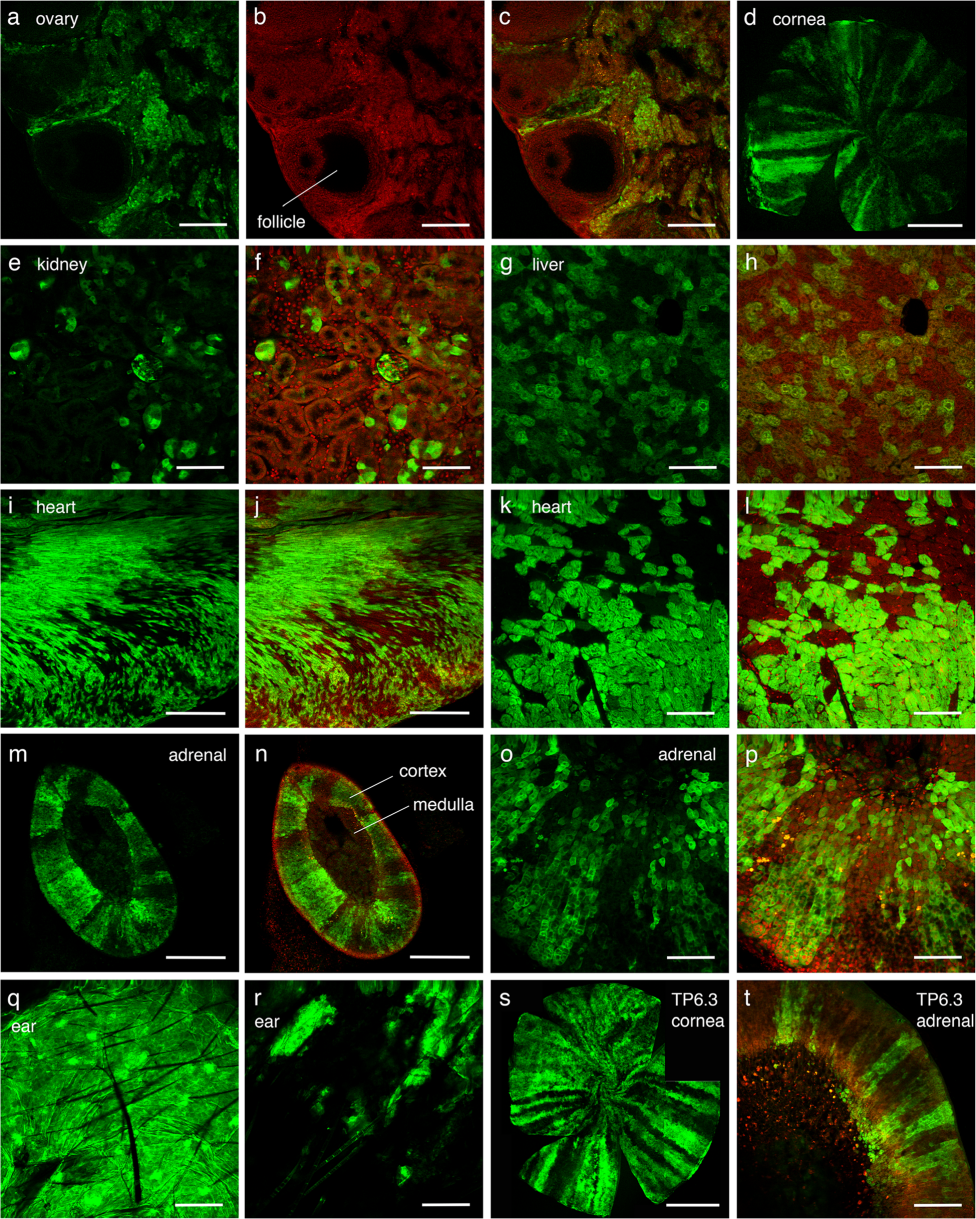
Non-ubiquitous and mosaic tauGFP expression. **a-c** Confocal images of vibratome section of adult tauGFP-positive *TgTP6.4* ^*Tg/−*^ ovary showing that the transgene is expression in some ovarian cell types but not in the follicular granulosa cells: **a** tauGFP fluorescence, (**b**) propidium iodide (PI) counterstain and (**c**) combined tauGFP and PI image. **d** Flat mount of *TgTP6.4* ^*Tg/−*^ cornea showing mosaic tauGFP expression. **e-p** Confocal images of vibratome sections of tauGFP fluorescence (**e**, **g**, **i**, **k**, **m**, **o**) and combined tauGFP fluorescence and PI counterstain (**f**, **h**, **j**, **l**, **n**, **p**), showing mosaic tauGFP expression in different *TgTP6.4* ^*Tg/−*^ tissues and organs: (**e**, **f**) kidney, (**g**, **h**) liver, (**i**-**l**) heart and (**m**-**p**) adrenal cortex. **q**, **r** Confocal images of whole mounts of ear punch biopsies from *TgTP6.4* ^*Tg/−*^ mice showing (**q**) ubiquitous and (**r**) mosaic tauGFP expression. **s**, **t** Mosaic tauGFP expression in two *TgTP6.3* ^*Tg/−*^ tissues: (**s**) montage of flat mount of cornea and (**t**) vibratome section of adrenal cortex. Scale bars: **e**, **f**, **g**, **h**, **k**, **l**, **o**, **p** = 100 μm; **a**, **b**, **c**, **q**, **r**, **t** = 200 μm; **i**, **j**, **m**, **n** = 500 μm; **d**, **s** = 1000 μm

**Fig. 5:**
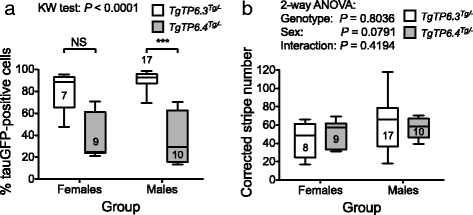
Comparison of percentage of tauGFP-positive cells and corrected stripe number in mosaic adrenal cortices from four groups of tauGFP transgenic mice. **a** *TgTP6.3* ^*Tg/−*^ mosaic adrenal cortices had a higher mean percentage of tauGFP-positive cells than *TgTP6.4* ^*Tg/−*^ mosaic cortices and, for males, this was significant by Kruskal-Wallis (KW) tests and Dunn’s multiple comparison post-tests. There were no significant differences between males and females of the same genotype. The mean percentages of tauGFP-positive cells (±95% CI) were: *TgTP6.3* ^*Tg/−*^ females, 81.1 ± 12.4%; *TgTP6.4* ^*Tg/−*^ females, 38.5 ± 13.1%; *TgTP6.3* ^*Tg/−*^ males, 91.0 ± 3.3%; *TgTP6.4* ^*Tg/−*^ males, 37.7 ± 14.7%. **b** There were no significant differences in corrected stripe numbers in mosaic adrenal cortices among any of the four groups of mice (both sexes for two genotypes) by 2-way ANOVA and Bonferroni’s multiple comparison post-tests. The mean corrected stripe numbers (±95% CI) were: *TgTP6.3* ^*Tg/−*^ females, 44.1 ± 12.8; *TgTP6.4* ^*Tg/−*^ females, 50.8 ± 9.5; *TgTP6.3* ^*Tg/−*^ males, 60.5 ± 12.2; *TgTP6.4* ^*Tg/−*^ males, 57.0 ± 7.2. The box and whisker plots show the median (horizontal line within the box), upper and lower quartiles (top and bottom of boxes), and the minimum and maximum of all the data (ends of whiskers). ***P < 0.001. The number of adrenal glands in each group is shown within or above the box and whiskers; one adrenal gland was analysed per mouse

C57BL/6 and outbred CD-1 mice were purchased from Bantin and Kingman, Hull, UK and A/J/Ola/Hsd mice were purchased from Harlan Olac Ltd., Bicester, UK. BALB/c/Eumm, CBA/Ca, C57BL/OlaWs (a C57BL/OlaHsd sub-colony), (C57BL × CBA/Ca)F1 hybrids, TP6.3 and TP6.4 mice were bred and maintained under conventional conditions at the University of Edinburgh with a light cycle of 14 h light (05:00 h. - 19:00 h.) and 10 h dark or 12 h light (06:00 h. - 18:00 h.) and 12 h dark. Founder TP6.3 and TP6.4 mice were produced from chimaeras made with E14Tg2a ES cells that had been transfected with the CAG-tauGFP vector, pTP6 [1, 2]. Founder mice were on a largely outbred MF1 genetic background and the transgenes were bred onto other strains as required. Transgenic mice were maintained by crossing *TgTP6.3* ^*Tg/−*^ and *TgTP6.4* ^*Tg/−*^ hemizygotes to non-transgenic, wild-type (WT) mice. Both TP6.3 and TP6.4 mice were initially crossed to inbred C57BL/6 mice to produce the first colony of each transgenic line, and some mice were also crossed to outbred, albino CD-1 mice to facilitate confocal microscopy of tauGFP expression in eye tissues. These colony I mice were used for the initial investigations shown in Fig. 3 and Additional file 1: Table S1. For later studies, *TgTP6.3* ^*Tg/−*^ and *TgTP6.4* ^*Tg/−*^ mice from the first colonies were crossed to (C57BL × CBA/Ca)F1 hybrid mice to produce a second colony for each line. These colony II mice were maintained by crossing transgenic mice to (C57BL × CBA/Ca)F1 mice at each generation and were used for the investigations shown in Figs. 1, 2, 4, 5, Additional file 1: Figures S2, S3 and S4 and Table 1. In all genetic crosses the female parental genotype is shown first. TauGFP-positive offspring were identified at weaning by the green fluorescence of their ear punch biopsies, using a fluorescence microscope with a fluorescein isothiocyanate (FITC) filter set. Some tauGFP-positive newborn pups were identified as described elsewhere [13].

### Analysis of postnatal growth

Newborn pups from crosses between (C57BL × CBA/Ca)F1 females and hemizygous *TgTP6.4* ^*Tg/−*^ males were sexed and weighed on postnatal days (P) 1 and 7 and then at weekly intervals thereafter until P84. Individual pups were marked with a marker pen until they were old enough for an ear punch biopsy, which was used both for identification and classification of their tauGFP phenotype.

### Analysis of preimplantation embryos

For collection of preimplantation embryos, females were superovulated by intraperitoneal (i.p.) injections of 5 international units (I.U.) of pregnant mare’s serum gonadotrophin (PMSG; Folligon, Intervet) at approximately 12 noon followed 48 h later by 5 I.U. of human chorionic gonadotrophin (hCG; Chorulon, Intervet). After hCG injections, each female was caged with a male overnight and mating was verified by the presence of a vaginal copulation plug the following morning, which was designated embryonic day (E) 0.5. Preimplantation embryos were flushed from the reproductive tract with KSOM-H handling medium [14] and their ages in hours post coitum (p.c.) were timed from the mid-point of the dark period (midnight) following the hCG injection. For collection of 1-cell stage embryos, mice were killed at approximately 10:00 h. on the morning after the hCG injection, cumulus masses were released from the oviducts and cumulus cells were dispersed in a solution of 100 I.U. hyaluronidase (Sigma-Aldrich, Gillingham, UK) per ml of phosphate-buffered saline (PBS). Two cell-stage embryos were flushed from the oviducts at approximately 10:00 h. on the following day (34 h.).

For time-lapse monitoring, preimplantation embryos were cultured for 24 h in drops of KSOM culture medium under mineral oil in thin glass-bottomed dishes (WillCo HBSt 3522, Intracel Ltd. Royston, UK). The dishes were placed in an environmental chamber on top of the heated stage (THD 60, Linkam Scientific Instruments Ltd., Tadworth, UK) of an inverted Leica DMIRB/E confocal microscope, and the atmosphere within the chamber was maintained at 37 °C, 5% CO_2_ in air (Additional file 1: Fig. S1). Images were acquired every 15 or 30 min for approximately 24 h. in both fluorescent (FITC) and transmitted light mode, using the Leica TCS NT confocal system. The time-lapse files contained both FITC and transmitted light channels from each time point. These were merged to provide photographs showing GFP fluorescence overlaid on a transmitted light image for the figures. Each image from the FITC channel is a pseudocoloured greyscale representation of the intensity of the emission signal from the sample, rendered on a 0–255 pixel intensity scale, which represents the strength of fluorescence. For quantitative analysis, files for each time point were opened in the freeware image analysis program, Scion Image (http://downloads.informer.com/scion-image/), each embryo was outlined as a region of interest (ROI) and an average pixel intensity reading obtained. If embryos moved between frames (time points), the ROI was either moved or redrawn for analysis of the next frame. Embryos that were cultured from the 2-cell stage but failed to cleave were excluded from the analysis. The embryos sometimes drifted out of focus so, typically, a short (1–2 h.) culture period was recorded at the beginning of the experiment and examined, so the focus could be adjusted if required. The focus was also checked prior to overnight recording. These interruptions meant that each time-lapse experiment comprised several separate files, which sometimes differed slightly in pixel intensity because of fluctuations in laser power that occurred while scanning was interrupted.

### Analysis of transgenic fetuses

Fetuses were produced by natural matings and staged from the date of the copulation plug, as described above. Females were killed by cervical dislocation, and the numbers of implantation sites, moles (resorbing conceptuses), dead fetuses and live fetuses were noted. Conceptuses were removed from the uterus and the fetuses dissected free of their placentas and extraembryonic membranes with fine forceps. Fetuses were classified for tauGFP expression with a Leica M2FLIII, fluorescence dissecting microscope using a GFP filter set. For comparison of fetal sizes, E14.5 fetuses were blotted dry, weighed and their crown-rump lengths were measured.

### Analysis of tauGFP expression in tissue sections from fetal and adult transgenic mice

Hemizygous adults and E13.5 and E17.5 fetuses were produced as described above. Fetuses were decapitated and fixed in ice cold 4% paraformaldehyde (PFA) in phosphate buffered saline (PBS) overnight at 4 °C. Some small tissue samples from adults were also fixed by this immersion method. Other adult samples were perfusion fixed, while the mouse was deeply anaesthetised with a 0.4 ml i.p. injection of 25% urethane in saline. The vascular system was then flushed with ice-cold 0.9% saline via cardiac puncture, followed by perfusion with ice-cold 4% PFA in PBS. After perfusion, the mouse was killed and various organs, including brain, heart, liver, kidney and eyes, were removed and fixed overnight in fresh ice-cold 4% PFA in PBS.

Fixed samples were washed in two changes of PBS and embedded in 4% agarose (LMP Agarose, Bethesda Research Laboratories, Life Tech inc., USA). Blocks were trimmed, mounted on glass with adhesive and 200 μm sections were cut with a vibratome. Prior to mounting, some vibratome tissue sections were counterstained with propidium iodide (PI) solution (0.1% PI, 0.05% RNase, 0.1% Triton X-100 in PBS) for 5 min then rinsed in PBS, to label nuclear DNA fluorescent red. In some cases RNase was omitted, so cytoplasmic RNA was also counterstained. Sections were washed in PBS, immersed in a mixture of glycerol and PBS (1:1 v/v) at 4 °C until saturated and mounted on glass slides in a 1:1 mixture of glycerol and PBS containing 1% Vectashield antifade reagent (H-100, Vector Labs Inc., USA). The coverslips were sealed with Aquamount. Sections were viewed with an upright Leica TCS NT confocal microscope (Leica Microsystems, Germany). Bright-field images were collected in the transmitted light channel and GFP was detected in the FITC channel. For sections that were counterstained with propidium iodide, optical sections were acquired simultaneously in the FITC (green) and tetramethylrhodamine isothiocyanate (TRITC; red) channels. Single optical sections and stacks of sections were acquired with a minimum of 4 averages per optical section.

### Analysis of tauGFP expression by fluorescence activated cell sorting

TauGFP-positive and negative E14.5 fetuses, from crosses to outbred CD-1 strain mice, were dissected in ice-cold Earle’s Balanced Salt Solution (EBSS), pre-equilibrated in 5% CO_2_. Whole brains, hippocampus, cerebral cortex, ventral telencephalon, central and dorsal thalamus, and midbrain and hindbrain tissues were dissected and dissociated using the Worthington Papain Dissociation System, (Worthington Biochemical Corporation, USA). Dissociated cells were sorted for green fluorescence using a Fluorescence Activated Cell Sorter (FACS; Becton Dickinson, Rutherford, NJ, USA) to generate histograms of green fluorescence intensity (FL1 channel) versus cell number for 10,000 cells per sample. Gates identifying cell populations with different fluorescent intensities were defined using a tauGFP-positive TP6.3 brain sample, considered to contain 100% fluorescent cells and the same gates were applied to all samples. After gating to exclude outliers (predominantly dead cells), the percentage of cells in three gated regions, corresponding to different fluorescent intensities, were compared: region M1 included all fluorescent cells, M2 included cells with low fluorescence and M3 included cells with high fluorescence.

### Analysis of mosaicism in adrenal glands

In *TgTP6.4* ^*Tg/−*^ and many *TgTP6.3* ^*Tg/−*^ hemizygous mice, the adrenal cortex was not uniformly tauGFP-positive but, in sections, showed a radial pattern of tauGFP-positive and tauGFP-negative stripes, most of which extended continuously across the full width of the adrenal cortex. This 2-dimensional radial stripe pattern in sections was analysed as a 1-dimensional pattern of stripe widths by measuring around the adrenocortical circumference as described previously [15]. The width of each tauGFP-negative and tauGFP-positive stripe was measured in calibrated images of single sections near the middle of the adrenal gland using UTHSCSA Image Tool software for Microsoft Windows (http://compdent.uthscsa.edu/dig/itdesc.html). Measurements were made around the complete circumference of the adrenal cortex at a similar distance from the outer edge of the adrenal gland (equivalent to 20% of the distance from the capsule to the inner margin of cortex). This provided the numbers of tauGFP-negative and tauGFP-positive stripes, the observed mean stripe widths, the total measured circumference and the proportions of tauGFP-negative and tauGFP-positive cells at the depth of the measurements for each adrenal cortex analysed. To allow for differences in stripe widths at different radial positions, the stripe width was expressed as a proportion of the circumference.

The radial stripes are believed to represent coherent clones of cells derived from stem cells at the periphery of the adrenal cortex [15, 16]. However, it is misleading to compare observed stripe numbers directly because each stripe may comprise several adjacent coherent clones and the number of clones per stripe is likely to vary according to the proportions of tauGFP-positive and tauGFP-negative cells in the tissue. We, therefore, converted the observed number of tauGFP-positive stripes plus tauGFP-negative stripes to a ‘corrected stripe number’ for each section, which adjusts for the expected number of clones per stripe [15]. First, the observed mean tauGFP-negative stripe width was divided by the correction factor 1/(1-p), where p is the proportion of tauGFP-negative cells around the circumference [15, 17, 18]. As the radial stripes in sections of the adrenal cortex form a complete circle, the calculated ‘corrected mean stripe width’ will be the same for both the positive and negative stripes, as explained previously [15, 17, 18]. Thus, correction of mean tauGFP-positive stripe widths, using the proportion of tauGFP-positive cells as p, produced identical results. As the ‘corrected mean stripe width’ applies to both tauGFP-negative and tauGFP-positive stripes, the ‘corrected stripe number’ for the positive plus negative stripes is the reciprocal of the corrected mean stripe width, expressed as the proportion of the measured circumference. The calculated corrected stripe number adjusts for differences in proportions of tauGFP-positive cells among adrenals and is likely to be proportional to the number of active coherent clones of stem cells, so was used to compare different groups of adrenal glands as described elsewhere [15].

### Statistics

Minimum group sizes were guided by previous experience and power calculations. For comparison of postnatal growth, TP6.4 group sizes were chosen to ensure sufficient power to detect, as significant (*P* < 0.05), mean body mass differences that were smaller than those previously published for TP6.3 [1, 2]. For quantitative analysis of adrenal mosaicism, results for only one adrenal gland per mouse were included. For mice where both adrenal glands were imaged, the choice of including the left or right adrenal was randomised by tossing a coin. The choice of parametric or non-parametric tests was guided, in part, by normality tests. GraphPad Prism versions 5.0c and 7 (GraphPad Software Inc., La Jolla, CA) were used for most statistical tests including 2-way analysis of variance (ANOVA) followed by Bonferroni multiple comparison post-tests, repeated measures 2-way ANOVA followed by Bonferroni multiple comparison post-tests, 1-way ANOVA, the paired t-test, the Mann–Whitney U-test and the Kruskal-Wallis test followed by Dunn’s multiple comparison post-tests. An online statistical calculator (http://vassarstats.net/index.html) was used for the chi-square goodness of fit test and Fisher’s exact test. Error bars shown are 95% confidence intervals (CI).

## Results

### Postnatal growth and viability of hemizygous *TgTP6.4*^*Tg/−*^ transgenic mice

Figure 1a, b shows postnatal growth curves, for offspring from crosses between (C57BL × CBA/Ca)F1 females and hemizygous, *TgTP6.4* ^*Tg/−*^ males. The hemizygous males were themselves produced from crosses with (C57BL × CBA/Ca)F1 mice, so at least 75% of the genetic background of the weighed offspring was from the C57BL and CBA/Ca strains. Results showed that tauGFP-positive male offspring were significantly lighter than tauGFP-negative males from postnatal day (P) 28 but that tauGFP-positive females grew normally. Nevertheless, growth was less severely affected in tauGFP-positive, TP6.4 males than in tauGFP-positive, TP6.3 males or females. For comparison, previously published results for TP6.3 mice [2] are shown in Fig. 1c,d. Four mice from the TP6.4 cross died before the end of the growth experiment but postnatal deaths were not more common among tauGFP-positive offspring. The surviving mice comprised 45 tauGFP-negative females, 50 tauGFP-positive females, 61 tauGFP-negative males and 47 tauGFP-positive males (Fig. 1a,b). This frequency distribution did not differ from the expected 1:1:1:1 ratio (*P* = 0.3901 by goodness of fit chi-square test) and the overall frequency of tauGFP-positive mice (97/203 = 47.8%) was not significantly different from 50% (*P* = 0.5716), implying that viability of *TgTP6.4* ^*Tg/−*^ hemizygotes from this cross was normal. These crosses also provide evidence that the *TgTP6.4* insertion site is not on the X-chromosome (unless it is in the pseudoautosomal region) because tauGFP-positive males transmitted the transgene approximately equally to their sons and daughters.

### Viability of *TgTP6.4*^*Tg/Tg*^ homozygotes

We were unable to identify any adult *TgTP6.4* ^*Tg/Tg*^ homozygotes by overt differences in GFP fluorescence and, as the *TgTP6.4* insertion site is unknown, we had no simple PCR method for distinguishing *TgTP6.4* ^*Tg/Tg*^ homozygotes from *TgTP6.4* ^*Tg/−*^ hemizygotes. In the absence of an easy method for identifying homozygotes, we used a statistical analysis of offspring phenotype frequencies from a genetic test cross to determine whether homozygotes were viable. Thirty-nine tauGFP-positive progeny, from *TgTP6.4* ^*Tg/−*^ × *TgTP6.4* ^*Tg/−*^ crosses, were crossed to non-transgenic mice to determine whether any only produced tauGFP-positive offspring and so were likely to be homozygotes. Thirty-six mice were classified as hemizygotes because they produced both tauGFP-positive and tauGFP-negative offspring in either their first viable litter (35 cases) or their second litter (one case). One other mouse produced three litters with 9/9, 7/8 and 9/12 tauGFP-positive offspring, respectively. As the overall frequency (25/29) was significantly higher than the expected 50% tauGFP-positive offspring (*P* = 0.0002), this mouse might have been a germline homozygous/hemizygous mosaic, rather than a hemizygote but it died before further investigations could be performed. Five of the other 36 mice produced equal numbers of tauGFP-positive and tauGFP-negative offspring, 16 produced more tauGFP-positive and 15 produced more tauGFP-negative offspring. As the 16:15 ratio did not differ significantly from the 1:1 ratio expected if all 36 mice were *TgTP6.4* ^*Tg/−*^ hemizygotes (*P* = 1.0000), there was no evidence that any of these were homozygous/hemizygous mosaics. This is also supported by the overall frequency of tauGFP-positive offspring from these 36 mice (177/345; 51.3%), which was not significantly different from the expected 50% (*P* = 0.6714) for hemizygotes. The remaining two males failed to sire any offspring in three-months and we cannot exclude the possibility that these were infertile homozygotes. One third of the tauGFP-positive offspring from *TgTP6.4* ^*Tg/−*^ × *TgTP6.4* ^*Tg/−*^ crosses are expected to be homozygous but none of 37 fertile offspring bred as homozygotes. The probability of this occurring by chance is only (^2^/_3_)^37^ (*P* = 0.0000003), if homozygotes are actually viable and fertile. These results imply that homozygous *TgTP6.4* ^*Tg/Tg*^ mice do not survive to reproduce.

Further statistical evidence that homozygotes do not survive to adulthood was obtained retrospectively from our mouse colony breeding records for three crosses. Offspring were classified as tauGFP-positive or tauGFP-negative by fluorescence microscopy of ear tissue biopsied, when they were weaned at about 3 weeks (Table 1). As the maternal genotype has the potential to affect embryonic development, cross 2 (*TgTP6.4* ^*Tg/−*^ female × WT, *TgTP6.4* ^*−/−*^ male) is the most appropriate control for the experimental cross (*TgTP6.4* ^*Tg/−*^ × *TgTP6.4* ^*Tg/−*^). The percentage of tauGFP-positive mice in this control cross (43.5%) did not differ significantly from the expected 50% but it was significantly lower in the reciprocal cross (39.3%; see Table 1 for statistical analysis). This was unexpected, as the equivalent WT, *TgTP6.4* ^*−/−*^ female × *TgTP6.4* ^*Tg/−*^ male cross produced 47.8% tauGFP-positive mice for the growth curve experiment (Fig. 1). However, the retrospective breeding data were collected after the growth experiment was completed and records of later generations showed that many tauGFP-positive offspring had a mosaic tauGFP phenotype. Consequently, the tauGFP-positive frequency might have been underestimated if some *TgTP6.4* ^*Tg/−*^ offspring, with mosaic transgene expression, were misclassified as tauGFP-negative from their ear punch biopsies. The percentage of tauGFP-positive mice in the experimental cross (55.9%) was significantly below 75% (Table 1), suggesting that homozygotes do not survive until 3 weeks even if this frequency was also slightly underestimated. Furthermore, significantly more pups died between birth and weaning in the experimental than the control cross (Table 1) and death of 13.6% of the pups in the experimental cross was attributed to differences between the crosses. As this is less than the 25% expected, if all homozygotes died, some may die before birth or too soon after birth to be recorded.

**Table 1:** Viability of *TgTP6.4* hemizygotes and homozygotes assessed by frequencies of tauGFP-positive mice produced in different crosses

	Genetic cross (female × male)		
Progeny	Cross 1 (experimental cross)	Cross 2 (control cross)	Cross 3
	*Tg/−* × *Tg/−*	*Tg/−* × *−/−*	*−/−* × *Tg/−*
A. Mice classified for tauGFP at ~3 weeks			
Total born	336	140	348
Dead before weaning	52 (15.5%)^a^	3 (2.1%)	8 (2.3%)
Dead after weaning (most unclassified)	5 (1.5%)	0	3 (0.9%)
Viable at weaning	284	137	340
TauGFP not classified	46	6	139
TauGFP classified	238	131	201
TauGFP-positive	133 (55.9%)^b,c^	57 (43.5%)^d^	79 (39.3%)^e^
TauGFP-negative	105 (44.1%)	74 (56.5%)	122 (60.7%)
Pre-weaning death attributable to cross^j^	13.6%	NA	NA
B. Fetuses classified for tauGFP at E14.5			
Total conceptuses	193	192	
Moles (early deaths)	16 (8.3%)^f^	9 (4.7%)	
Dead fetuses	2 (1.0%)^f^	2 (1.0%)	
Total live fetuses	175	181	
TauGFP-positive	129 (73.7%)^g,h^	85 (47.0%)^i^	
TauGFP-negative	46 (26.3%)	96 (53.0%)	
Death attributable to cross^k^	3.8%	NA	

*Abbreviations*: *−/−* non-transgenic *TgTP6.4* ^*−/−*^ was wild-type, (C57BL × CBA/Ca)F1, *Tg/−* hemizygous *TgTP6.4* ^*Tg/−*^, *NA* not applicable.

In (A), data were compiled retrospectively from breeding records and not all offspring were classified. Data were excluded if the whole litter died between birth and weaning or no offspring were classified. In later litters it was recorded that tauGFP expression was mosaic in ear tissue of some tauGFP-positive offspring. ^a^The frequency of death before weaning was significantly higher in cross 1 than cross 2 (*P* < 0.0001 by Fisher’s exact test). ^b-e^The proportion of tauGFP-positive progeny in cross 1 differed significantly by goodness of fit chi-square test from 75% expected, if all homozygotes and hemizygotes are viable (*P* < 0.0001)^b^, and from 67% expected, if all homozygotes die but all hemizygotes are viable (*P* = 0.0005)^c^. The proportion of tauGFP-positive progeny did not differ significantly from 50% expected in cross 2 (*P* = 0.1615 by goodness of fit chi-square test)^d^ but it did differ from this expected proportion in cross 3 (*P* = 0.003)^e^. ^f^ In (B), the frequencies of moles plus dead fetuses did not differ significantly between crosses 1 and 2 (*P* = 0.2463 by Fisher’s exact test). ^g-i^ The proportion of tauGFP-positive fetuses in cross 1 did not differ significantly by goodness of fit chi-square test from either 75% expected, if all homozygotes and hemizygotes are viable (*P* = 0.7642)^g^ or from 67% expected, if all homozygotes die but all hemizygotes are viable (*P* = 0.0578)^h^. In cross 2, the proportion of tauGFP-positive progeny did not differ significantly from 50% expected (*P* = 0.4543 by goodness of fit chi-square test)^i^

The percentage death, attributable to production of *TgTP6.4* ^*Tg/Tg*^ homozygotes in cross 1, was calculated by correcting for sporadic death in cross 2 [61]:

^j^ $$ \left[1-\left(\frac{\mathrm{viable}\ \mathrm{mice}\ \mathrm{weaned}\ \mathrm{in}\ \mathrm{experimental}\ \mathrm{cross}\ 1}{\mathrm{total}\ \mathrm{born}\ \mathrm{in}\ \mathrm{experimental}\ \mathrm{cross}\ 1}/\frac{\mathrm{viable}\ \mathrm{mice}\ \mathrm{weaned}\ \mathrm{in}\ \mathrm{control}\ \mathrm{cross}\ 2}{\mathrm{total}\ \mathrm{born}\ \mathrm{in}\ \mathrm{control}\ \mathrm{cross}\ 2}\right)\right]\times 100 $$

^k^ $$ \left[1-\left(\frac{\mathrm{live}\ \mathrm{fetuses}\ \mathrm{in}\ \mathrm{experimental}\ \mathrm{cross}\ 1}{\mathrm{total}\ \mathrm{conceptuses}\ \mathrm{in}\ \mathrm{experimental}\ \mathrm{cross}\ 1}/\frac{\mathrm{live}\ \mathrm{fetuses}\ \mathrm{in}\ \mathrm{control}\ \mathrm{cross}\ 2}{\mathrm{total}\ \mathrm{conceptuses}\ \mathrm{in}\ \mathrm{control}\ \mathrm{cross}\ 2}\right)\right]\times 100 $$

To investigate whether homozygous, *TgTP6.4* ^*Tg/Tg*^ E14.5 fetuses were viable, we compared observed and expected frequencies of tauGFP-positive fetuses in two crosses (Table 1). The control and experimental crosses produced close to 50 and 75% tauGFP positive E14.5 fetuses respectively, as expected if all hemizygotes and homozygotes survived to E14.5. Survival of most homozygotes to E14.5 is also suggested by the relatively low frequency of dead embryos, which was not significantly elevated in the experimental cross. However, there was some evidence that homozygous tauGFP fetuses were slightly smaller than normal by E14.5. After allowing for variation among litters, tauGFP-positive fetuses in the experimental cross were significantly lighter than tauGFP-negative fetuses (*P* < 0.0001 by a 2-way ANOVA) and had shorter crown-rump lengths (*P* < 0.0001; Additional file 1: Figure S2). In the control cross, fetal mass differences were less pronounced (*P* = 0.0343) and crown-rump length differences were not significant (*P* = 0.0597), implying that hemizygous *TgTP6.4* ^*Tg/−*^ fetuses were more normal in size at E14.5. In summary, the breeding experiments indicate that most *TgTP6.4* ^*Tg/Tg*^ homozygotes survive to E14.5 but some may already be retarded and they probably all die between E14.5 and weaning.

### TauGFP expression in *TgTP6.4*^*Tg/−*^ preimplantation embryos

To determine when the *TgTP6.4* transgene was first expressed, preimplantation embryos produced by (C57BL × CBA/Ca)F1 females and hemizygous *TgTP6.4* ^*Tg/−*^ males were collected, cultured in an environmental chamber on the stage of an inverted confocal microscope (Additional file 1: Figure S1) for up to 24 h. and imaged by time-lapse confocal microscopy to determine when tauGFP fluorescence became detectable. Fluorescence was not detected in embryos that were cultured from approximately 10 h. post coitum (p. c.) at the 1-cell stage (Fig. 2a) but it was detected in some embryos that were cultured from approximately 34 h. p.c. at the 2-cell stage (Fig. 2b,c). A plot of pixel intensities is shown in Fig. 2g for one group of embryos that were cultured from 34.5 h. (2-cell stage) to 56.5 h. (4-cell stage). This confirmed that tauGFP expression was detectable in *TgTP6.4* ^*Tg/−*^ embryos at the 2-cell stage and fluorescence increased during the 24 h. culture period. There was considerable variation in pixel intensity among *TgTP6.4* ^*Tg/−*^ embryos but there was no overlap with the tauGFP-negative *TgTP6.4* ^*−/−*^ embryos from 38.3 h. p.c. A repeated measures 2-way ANOVA with Bonferroni multiple comparison post-tests showed that pixel intensity first differed significantly between the two genotypes at 37.5 h. Although there were no significant differences before 37.5 h., some individual embryos that were subsequently identified as *TgTP6.4* ^*Tg/−*^ had higher pixel intensities than any *TgTP6.4* ^*−/−*^ embryos at the beginning of the experiment (34.5 h.). Some tauGFP-positive embryos were also identifiable visibly at this time (Fig. 2b) and differences probably reflect developmental heterogeneity. As the tauGFP fluorescence is detectable in 2-cell stage *TgTP6.4* ^*Tg/−*^ embryos by 37.5 h., and earlier in some embryos, the transgene will be expressed slightly before this age.

Preimplantation embryos from the reciprocal cross between *TgTP6.4* ^*Tg/−*^ females and (C57BL × CBA/Ca)F1 males, initially all retained oocyte-encoded tauGFP but *TgTP6.4* ^*Tg/−*^ and *TgTP6.4* ^*−/−*^ embryos could be distinguished by their tauGFP levels by the 4-cell stage (Fig. 2d-f). There was no overlap in pixel intensity from 45 h. p.c. for the experiment shown in Fig. 2h and a repeated measures 2-way ANOVA with Bonferroni multiple comparison post-tests showed that pixel intensity differed significantly between the two genotypes from 42.5 h., consistent with the onset of embryo-coded *TgTP6.4* expression by the late 2-cell stage. Comparisons of pixel intensity between *TgTP6.4* ^*−/−*^ embryos, with some residual tauGFP, and control WT embryos, with no tauGFP, showed no overlap in pixel intensity at any ages analysed (34.6 to 57.6 h.) and a repeated measures 2-way ANOVA with Bonferroni multiple comparison post-tests confirmed that pixel intensities remained significantly different at 57.6 h. This indicates that residual oocyte-encoded tauGFP remained at least until the 4-cell stage.

### TauGFP expression in fetal and adult *TgTP6.4*^*Tg/−*^ tissues

Confocal microscopy of E13.5 fetal heads showed that tauGFP fluorescence in the brain was weaker in *TgTP6.4* ^*Tg/−*^ than in *TgTP6.3* ^*Tg/−*^ hemizygotes (Fig. 3a,b) but the choroid plexus, which is rich in blood capillaries, fluoresced strongly (Fig. 3b), and other blood vessels in the *TgTP6.4* ^*Tg/−*^ fetal brain were also clearly delineated (Fig. 3c). In *TgTP6.4* ^*Tg/−*^ fetal eyes, most of the neural retina showed little tauGFP fluorescence but the nerve fibre layer and inner nuclear layer of the neural retina fluoresced weakly and the retinal pigment epithelium, blood vessels and particularly the lens fluoresced strongly (Fig. 3d-f). Many other *TgTP6.4* ^*Tg/−*^ fetal tissues showed high levels of fluorescence including heart and lung (Fig. 3g,h). Much of the adult *TgTP6.4* ^*Tg/−*^ brain was characterised by poor tauGFP fluorescence, similar to fetal brain, although the Bergmann glia and blood vessels of the cerebellum fluoresced more strongly (Fig. 3i-k). Levels of tauGFP fluorescence varied among and within other adult *TgTP6.4* ^*Tg/−*^ organs. Like other blood vessels, the capillary knots of the kidney glomeruli fluoresced particularly strongly (Fig. 3l) and fluorescence was also readily detectable in lung, heart and liver (Fig. 3m-p).

Fluorescence activated cell sorting (FACS) analysis of cell suspensions prepared from whole E14.5 fetal brains confirmed that tauGFP fluorescence was present in fewer *TgTP6.4* ^*Tg/−*^ cells than *TgTP6.3* ^*Tg/−*^ cells (Fig. 3q-t). The analysis using three gated regions (Fig. 3q) is described in the Methods. Fig 3r shows that 95% of all the cells from whole *TgTP6.3* ^*Tg/−*^ brains were fluorescent (M1 gate in Fig. 3q), of which 87% were highly fluorescent (Fig. 3r). In contrast, only 25% of cells from whole *TgTP6.4* ^*Tg/−*^ brains were fluorescent, 10% of which were highly fluorescent (Fig. 3s). Nevertheless, this profile shows more fluorescence than the *TgTP6.4* ^*−/−*^ negative controls (Fig. 3t). Similar results were obtained for cells from separate brain regions although the hippocampus showed more fluorescence than other *TgTP6.4* ^*Tg/−*^ brain regions (Additional file 1: Table S1).

The brain was not the only organ where *TgTP6.4* expression appeared to be restricted to specific tissues or regions. While tauGFP fluorescence was detected in some ovarian cell types in adult *TgTP6.4* ^*Tg/−*^ females, no fluorescence was seen in ovarian follicle granulosa cells or oocytes (Fig. 4a-c). Furthermore, although our initial survey of *TgTP6.4* ^*Tg/−*^ hemizygotes, on both predominantly C57BL/6 and outbred CD-1 backgrounds, showed that tauGFP was expressed relatively uniformly in most *TgTP6.4* ^*Tg/−*^ tissues, mosaic expression was increasingly common after several generations of crosses to (C57BL × CBA/Ca)F1 mice. Mosaicism affected all tissues examined, including the cornea, kidney, liver, heart and adrenal cortex (Fig. 4d-p). *TgTP6.4* ^*Tg/−*^ mice showing mosaic expression could be distinguished from those with uniform transgene expression by fluorescence microscopy of ear punch biopsies (Fig. 4q, r) and mice with a mosaic ear skin phenotype also showed mosaic expression in all internal organs examined. After several more generations, ear punch biopsies showed that all the *TgTP6.4* ^*Tg/−*^ mice in our colony had a mosaic expression pattern. In contrast, after crossing *TgTP6.3* ^*Tg/−*^ mice onto (C57BL × CBA/Ca)F1, we only detected mosaic expression in the cornea and adrenal cortex (Fig. 4s,t) and even this more restricted mosaicism only occurred in some mice. The *TgTP6.3* transgene appeared to be uniformly expressed in other tissues, as previously reported [1, 2].

### Comparison of mosaic tauGFP expression in *TgTP6.3*^*Tg/−*^ and *TgTP6.4*^*Tg/−*^ adrenal cortices

The pattern of radial stripes seen in the adrenal cortex of some *TgTP6.3* ^*Tg/−*^ and *TgTP6.4* ^*Tg/−*^ mice (Fig. 4m-p,t) has been reported for several other experimental systems and is believed to represent coherent clones of cells, which are derived from stem cells at the periphery of the adrenal cortex, and move towards the medulla (see Discussion). After correcting for the expected number of multiple adjacent clones in a single stripe, the corrected stripe number can provide an indirect estimate of the number of clones of active stem cells as described elsewhere [15, 17, 18] so this can be used to compare stem cell function in different groups. The uncorrected mean stripe width varies with the percentage of tauGFP-positive cells in the adrenal cortex, which will affect the number of clones per stripe, but the corrected mean stripe width adjusts for this, as shown in Additional file 1: Figure S3. The corrected mean stripe width was used to calculate a corrected stripe number, as explained in the Methods, to provide a quantitative comparison of *TgTP6.3* ^*Tg/−*^and *TgTP6.4* ^*Tg/−*^ stripe patterns.

We imaged vibratome sections of 60 adrenal glands, from 37 *TgTP6.3* ^*Tg/−*^ mice, 48 (80%) of which were mosaic (15 from 20 females and 33 from 40 males), and 31 adrenals, all of which were mosaic, from 20 *TgTP6.4* ^*Tg/−*^ mice (14 from 10 females and 17 from 10 males). Thus, at this stage, the frequency of adrenal mosaicism was high in both lines but it was significantly higher for *TgTP6.4* ^*Tg/−*^ (31/31 versus 48/60; *P* = 0.0069 by Fisher’s exact test). We analysed stripe patterns quantitatively for intact adrenal sections from mice that were at least 3 weeks old and grouped the results for one adrenal gland per mouse by genotype and sex. (After excluding adrenal glands from mice younger than 3 weeks, adrenal glands were analysed for 44 mice. Both adrenal glands were imaged for 18 mice but results were only included for one adrenal gland per mouse.) The mice differed widely in age but, as neither the percentage of tauGFP-positive cells nor the corrected mean stripe number differed significantly with age (Additional file 1: Figure S4), we pooled results for different ages within each of the four groups (males and females in the two transgenic lines). The results (Fig. 5) showed that, as well as having a higher frequency of mosaic adrenals, *TgTP6.4* ^*Tg/−*^ mice had mosaic adrenals with a lower percentage of tauGFP-positive cells (means were approximately 38% versus 86%). However, the quantitative analysis of stripe patterns showed that the corrected stripe number was similar for both genotypes, regardless of sex.

## Discussion

The aim of the present study was to determine whether the *TgTP6.4* marker transgene offered any advantages over *TgTP6.3*. Although tauGFP expression in *TgTP6.3* ^*Tg/−*^ cells provides an excellent marker, *TgTP6.3* ^*Tg/Tg*^ homozygotes do not survive and *TgTP6.3* ^*Tg/−*^ hemizygotes are slightly smaller than normal. *TgTP6.4* ^*Tg/−*^ growth was less affected than *TgTP6.3* ^*Tg/−*^ hemizygotes but *TgTP6.4* ^*Tg/Tg*^ homozygotes died between E14.5 and weaning age, as reported for *TgTP6.3* ^*Tg/Tg*^ homozygotes [2] and the widespread mosaicism means that *TgTP6.4* is unsuitable as a conventional cell lineage marker. The reason why both *TgTP6.3* ^*Tg/Tg*^ and *TgTP6.4* ^*Tg/Tg*^ homozygotes die was not investigated but high levels of GFP or tau protein are neurotoxic [19]. Other tauGFP transgenic mice have been produced by inserting a tauGFP transgene into a specific locus [20–22] or randomly inserting a CAG-tauGFP construct into the genome to produce another CAG-tauGFP transgenic line [23], but no mosaic phenotypes were described for these mice. While mosaicism in *TgTP6.3* ^*Tg/−*^ mice was only identified in the cornea and adrenal cortex, mosaicism became increasingly common in *TgTP6.4* ^*Tg/−*^ mice during our investigation and eventually affected all tissues examined and all *TgTP6.4* ^*Tg/−*^ mice.

Mosaic patterns produced by mouse chimaeras and X-inactivation mosaics have been useful for visualising clonal lineages and for analysing the extent of cell mixing and movement during development, growth and maintenance of adult tissues [24–29]. Mosaic transgene expression can provide a useful alternative for these types of investigations [15], particularly when mosaicism is widespread, as in *TgTP6.4* ^*Tg/−*^ mice. We used this approach to investigate whether there were any overt differences in maintenance of the adrenal cortex in *TgTP6.3* ^*Tg/−*^ and *TgTP6.4* ^*Tg/−*^ adults.

Mosaic *TgTP6.3* ^*Tg/−*^ and *TgTP6.4* ^*Tg/−*^ adrenals showed qualitatively similar radial stripes across the adrenal cortex. This pattern has previously been reported for other experimental systems, including mouse and rat chimaeras [24, 28, 30–33], mouse X-inactivation mosaics [33] and some other mouse lines that show mosaic transgene expression [15, 34–36]. The stripes are believed to represent coherent clones of cells that are derived from stem cells at the periphery of the adrenal cortex and they maintain the tissue as they move slowly across the cortex before undergoing apoptosis [15, 16, 37–40]. The corrected stripe numbers were similar in the two genotypes and were comparable to results reported previously for mouse chimaeras, X-inactivation mosaics and other mosaic transgenic mice [15, 33], suggesting that maintenance of the adrenal cortex involved a similar number of active clones of stem cells in all these groups.

Mosaic transgene expression is thought to involve position effect variegation caused by stochastic transgene silencing during development [34, 41–43]. Indirect evidence suggested that mosaic expression of a different transgene in the mouse adrenal cortex involved stochastic silencing at the level of the chromosome or transgene, rather than at the cellular level [44] but this may not apply to all mosaic transgenic lines. Transgene silencing can occur if the transgene integrates near an endogenous heterochromatic site [41], which may prevent the transcriptional machinery from accessing the transgene, or if it triggers the formation of heterochromatin by integrating as a series of repeated copies [45, 46]. Repeat-copy transgenes are also associated with methylation changes that could mediate stochastic transgene silencing if transgene methylation levels vary among cells [47]. Moreover, several genes that alter the extent of transgene silencing have been identified in a mutagenesis screen and some have been characterised [48, 49]. However, the copy number is not known for either of the transgenic lines that we studied and the mechanism(s) that caused widespread mosaic silencing of *TgTP6.4* and more restricted silencing of *TgTP6.3* is unknown.

TauGFP expression in *TgTP6.3* ^*Tg/−*^ preimplantation embryos was characterised previously [1, 2] and was similar in *TgTP6.4* ^*Tg/−*^ embryos. TauGFP fluorescence was ubiquitous in preimplantation embryos of both transgenic lines, so mosaic expression presumably involves transgene silencing in some cells during development. As there was no evidence for an age-effect on the percentage of cells expressing tauGFP in either *TgTP6.3* ^*Tg/−*^ or *TgTP6.4* ^*Tg/−*^ adrenals, we have no evidence that silencing is a continuous process. The simplest model assumes that transgene silencing involves a single stochastic event that occurs during development and the silent or active state is then stably inherited by daughter cells. As the frequency of *TgTP6.4* ^*Tg/−*^ mosaic expression changed over a number of generations of breeding and ultimately affected all tissues examined and the entire TP6.4 colony, it seems likely that the genetic background somehow affects the probability of transgene silencing but, again, the mechanism remains unknown.

In contrast to *TgTP6.4* ^*Tg/−*^ mice, mosaic expression did not occur in all *TgTP6.3* ^*Tg/−*^ mice, nor did it occur in so many tissues. Of the *TgTP6.3* ^*Tg/−*^ tissues examined, mosaicism was only identified in the adrenal cortex and cornea. Moreover, when mosaicism occurred in the adrenal cortex of *TgTP6.3* ^*Tg/−*^ mice, there were significantly more tauGFP-positive cells than in *TgTP6.4* ^*Tg/−*^ adrenals. This implies that silencing of the *TgTP6.3* transgene affects fewer cells per tissue as well as fewer tissues and fewer mice. In both *TgTP6.3* ^*Tg/−*^ and *TgTP6.4* ^*Tg/−*^ mice, the mosaic pattern in the cornea was in the form of radial stripes, strongly suggesting it was in the corneal epithelium layer, as described for studies of the corneal epithelium using other mosaic systems [17, 18, 50–57].

Mosaicism in *TgTP6.3* ^*Tg/−*^ mice is clearly more restricted than in *TgTP6.4* ^*Tg/−*^ hemizygotes so it might arise later in development but this was not tested directly. Investigation of why the extent of mosaicism differs between these two transgenic lines would probably require breeding both transgenes onto the same inbred genetic background, as well as knowledge of the insertion sites, copy numbers and epigenetic modifications around the insertion sites. Stem cells at the tissue’s periphery are thought to maintain both the adult corneal epithelium [55–60] and the adrenal cortex [15, 16, 38–40]. It may also, therefore, be worth investigating whether transgene silencing can occur in tissue-specific stem cells as well as earlier in development.

## Conclusion

Although widespread mosaic expression means that the *TgTP6.4* transgene is not a suitable alternative to *TgTP6.3* as a standard lineage marker, it provides a useful system for identifying and analysing clonal lineages. We exploited this mosaicism to demonstrate that similar clonal lineages maintained the adult adrenal cortex in different groups of mice. Differences, in the frequency and tissue distribution of mosaicism, between related transgenic lines, such as TP6.3 and TP6.4, may also provide a useful experimental system for investigating transgene silencing.

## Additional file

Additional file 1:
**Figure S1.** Confocal microscope with environmental chamber. (a) The Leica DMIRB/E inverted confocal microscope is shown together with the stage-mounted environmental chamber and some of the equipment required to maintain the correct temperature and gas phase within the chamber. (b) Side view of the environmental chamber with a culture dish on the heated stage. The flow of carbon dioxide from the gas cylinder to the environmental chamber was controlled by an infrared gas monitor to ensure that the gas mixture inside the environmental chamber was maintained at 5% CO2 in air. Embryos were cultured in drops of culture medium under liquid paraffin oil in a WillCo thin glass-bottomed culture dish on the heated stage. Time-lapse images of the embryos were acquired every 15 or 30 min in both fluorescent (FITC) and transmitted light modes for up to 24 h. Abbreviations: EC, environmental chamber; GC, gas cylinder; GM, gas monitor; HS, heated stage; HSC, heated stage controller. **Figure S2.** Comparison of sizes of E14.5 tauGFP-positive and tauGFP-negative fetuses from two crosses. (a-d) Comparisons of tauGFP-positive and tauGFP-negative fetal sizes using a 2-way analysis of variance (ANOVA) to allow for variation among litters. The graphs show fetal mass (a) and crown-rump length (b) for the *TgTP6.4* ^*Tg/−*^ female × *TgTP6.4* ^*Tg/−*^ male cross plus fetal mass (c) and crown-rump length (d) for the *TgTP6.4* ^*Tg/-*^ female × *TgTP6.4* ^*−/−*^ male cross. Each point represents a single fetus and fetuses are arranged by litters. Sexes were not distinguished and only litters with both tauGFP-positive and tauGFP-negative fetuses were included. Numbers of fetuses, means and *P*-values are shown. (e-h) Differences between within-litter means are shown for fetal mass and crown-rump length for tauGFP-positive and tauGFP-negative fetuses in each cross. *P*-values are shown for paired t-tests. Abbreviations: GFP-ve, tauGFP-negative; GFP + ve, tauGFP-positive. **Figure S3.** Relationship between mean stripe width and the percentage of tauGFP-positive cells in *TgTP6.4* ^*Tg/−*^ adrenal cortices. (a) The uncorrected mean tauGFP-positive stripe width for 27 *TgTP6.4* ^*Tg/−*^ adrenal glands varied widely. It was close to 2% of the adrenal circumference when the percentage of tauGFP-positive cells in the adrenal cortex was low but it was positively correlated with the percentage of tauGFP-positive cells. The Spearman correlation coefficient (r_s_) is shown. This positive correlation is likely to be because stripes may comprise several adjacent coherent clones of cells and the average number of tauGFP-positive clones per tauGFP-positive stripe will increase with the percentage of tauGFP-positive cells in the adrenal cortex. (b) The corrected mean tauGFP-positive stripe width was not significantly correlated with the percentage of tauGFP-positive cells so allows comparisons among adrenals with different percentages of tauGFP-positive cells. The observed (uncorrected) mean tauGFP-positive stripe number was corrected by dividing it by the correction factor 1/(1-p), where p is the proportion of tauGFP-positive cells around the circumference as explained in the Methods. **Figure S4.** Age has no effect on percentage of tauGFP-positive cells or corrected stripe number in mosaic adrenal cortices of *TgTP6.3* ^*Tg/−*^ and *TgTP6.4* ^*Tg/−*^ mice. (a-d) There were no significant differences among age groups for the % tauGFP-positive cells in the adrenal cortex for (a) *TgTP6.3* ^*Tg/-*^ females (b) *TgTP6.3* ^*Tg/-*^ males (c) *TgTP6.4* ^*Tg/-*^ females or (d) *TgTP6.4* ^*Tg/-*^ males. Mice were grouped into three or more age groups and analysed by the Kruskal-Wallis test (KW test). (e-h) There were also no significant positive correlations between age and the % tauGFP-positive cells in the adrenal cortex for any of the four groups. Spearman correlation coefficients (r_s_) are shown. (i-l) There were no significant differences among age groups for the corrected stripe number (tauGFP-positive stripes plus tauGFP-negative stripes) in the adrenal cortex for any sex and genotype combination. Mice were grouped into three or more age groups and analysed by the Kruskal-Wallis test (KW test) or 1-way ANOVA. (m-p) There were also no significant positive correlations between age and the corrected stripe number in the adrenal cortex for any sex and genotype combination. Pearson correlation coefficients (r) and the linear regression lines are shown but no lines differed significantly from horizontal. N = number of adrenal glands; one adrenal gland was analysed per mouse. **Table S1.** FACS analysis of fetal brains. (PDF 599 kb)
