# Supplementary material for: Comparison of two related lines of tauGFP transgenic mice designed for lineage tracing
Source: BMC Dev Biol. 2017 Jun 29;17:8. doi: 10.1186/s12861-017-0149-x (PMC5492368; doi:10.1186/s12861-017-0149-x)
Supplement: Additional file 1: — Figure S1. Confocal microscope with environmental chamber. (a) The Leica DMIRB/E inverted confocal microscope is shown together with the stage-mounted environmental chamber and some of the equipment required to maintain the correct temperature and gas phase within the chamber. (b) Side view of the environmental chamber with a culture dish on the heated stage. The flow of carbon dioxide from the gas cylinder to the environmental chamber was controlled by an infrared gas monitor to ensure that the gas mixture inside the environmental chamber was maintained at 5% CO2 in air. Embryos were cultured in drops of culture medium under liquid paraffin oil in a WillCo thin glass-bottomed culture dish on the heated stage. Time-lapse images of the embryos were acquired every 15 or 30 min in both fluorescent (FITC) and transmitted light modes for up to 24 h. Abbreviations: EC, environmental chamber; GC, gas cylinder; GM, gas monitor; HS, heated stage; HSC, heated stage controller. Figure S2. Comparison of sizes of E14.5 tauGFP-positive and tauGFP-negative fetuses from two crosses. (a-d) Comparisons of tauGFP-positive and tauGFP-negative fetal sizes using a 2-way analysis of variance (ANOVA) to allow for variation among litters. The graphs show fetal mass (a) and crown-rump length (b) for the TgTP6.4 Tg/− female × TgTP6.4 Tg/− male cross plus fetal mass (c) and crown-rump length (d) for the TgTP6.4 Tg/- female × TgTP6.4 −/− male cross. Each point represents a single fetus and fetuses are arranged by litters. Sexes were not distinguished and only litters with both tauGFP-positive and tauGFP-negative fetuses were included. Numbers of fetuses, means and P-values are shown. (e-h) Differences between within-litter means are shown for fetal mass and crown-rump length for tauGFP-positive and tauGFP-negative fetuses in each cross. P-values are shown for paired t-tests. Abbreviations: GFP-ve, tauGFP-negative; GFP + ve, tauGFP-positive. Figure S3. Relationship between mean stripe width and [file 12861_2017_149_MOESM1_ESM.pdf]

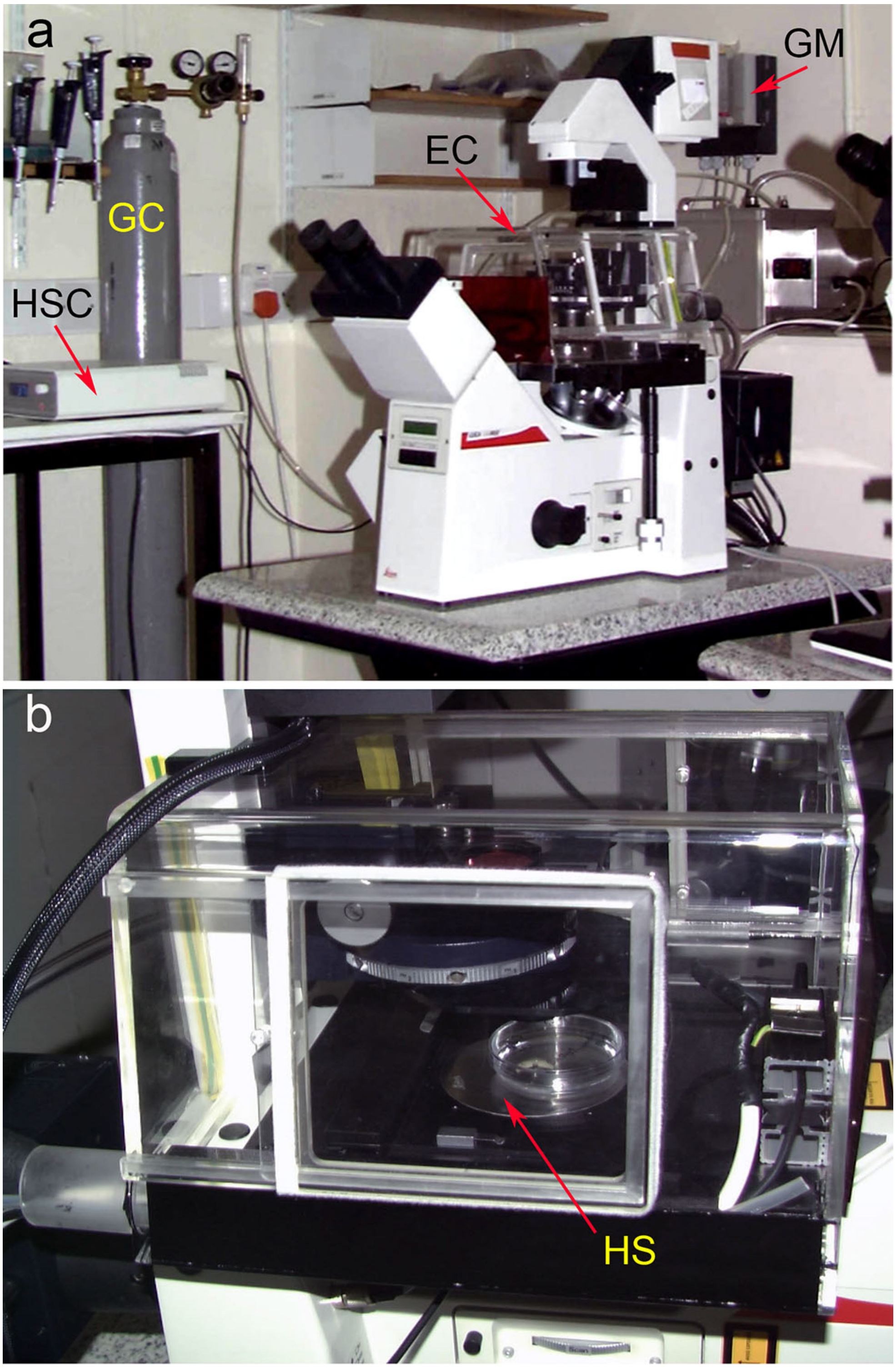

Figure S1. Confocal microscope with environmental chamber. (a) The Leica DMIRB/E inverted confocal microscope is shown together with the stage-mounted environmental chamber and some of the equipment required to maintain the correct temperature and gas phase within the chamber. **(b)** Side view of the environmental chamber with a culture dish on the heated stage. The flow of carbon dioxide from the gas cylinder to the environmental chamber was controlled by an infrared gas monitor to ensure that the gas mixture inside the environmental chamber was maintained at 5% CO_2 in air. Embryos were cultured in drops of culture medium under liquid paraffin oil in a WillCo thin glass-bottomed culture dish on the heated stage. Time-lapse images of the embryos were acquired every 15 or 30 minutes in both fluorescent (FITC) and transmitted light modes for up to 24 hours. Abbreviations: EC, environmental chamber; GC, gas cylinder; GM, gas monitor; HS, heated stage; HSC, heated stage controller.

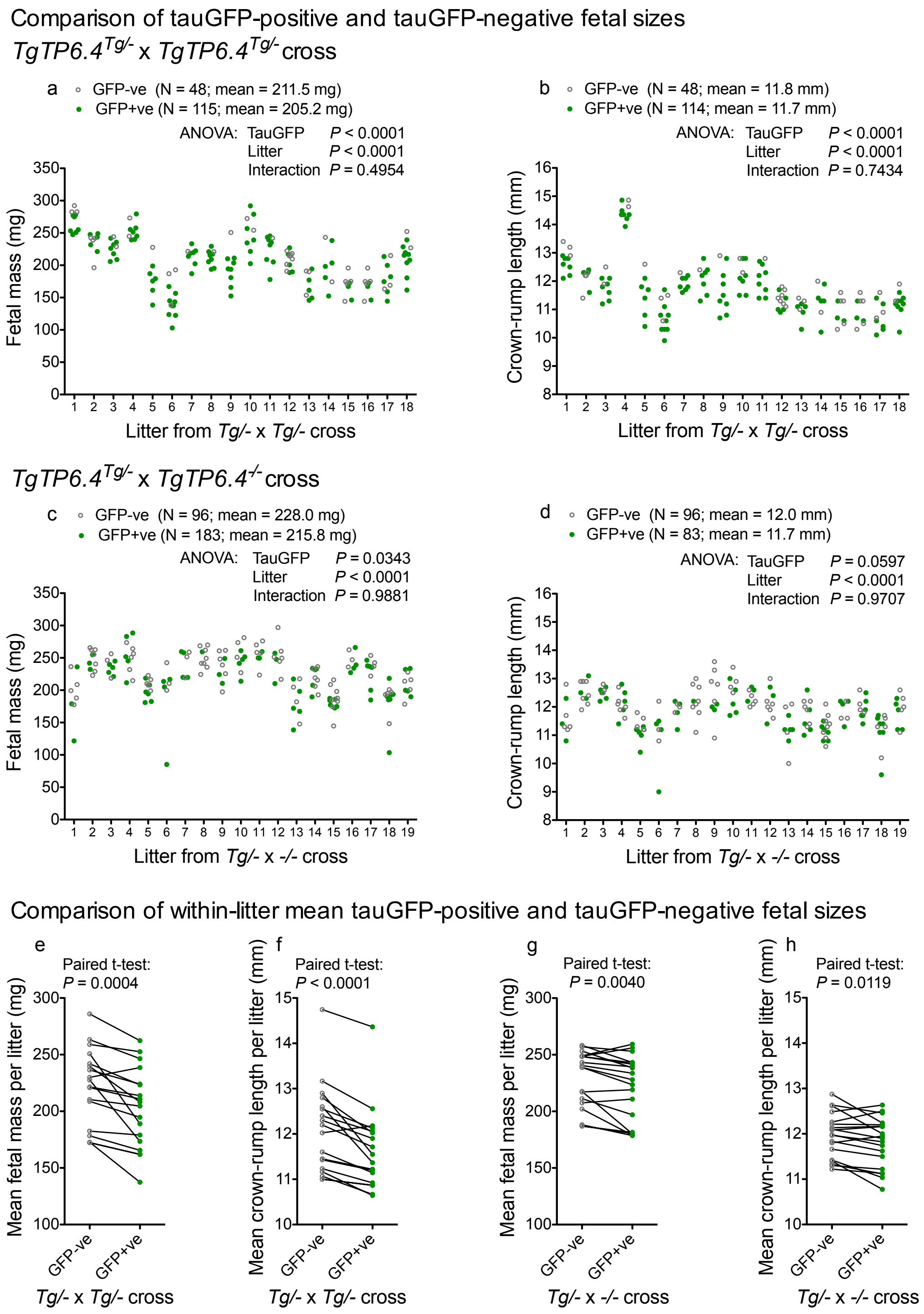

Figure S2. Comparison of sizes of E14.5 tauGFP-positive and tauGFP-negative fetuses from two crosses. (a-d) Comparisons of tauGFP-positive and tauGFP-negative fetal sizes using a 2-way analysis of variance (ANOVA) to allow for variation among litters. The graphs show fetal mass (a) and crown-rump length (b) for the *TgTP6.4*$^{Tg/-}$ female × *TgTP6.4*$^{Tg/-}$ male cross plus fetal mass (c) and crown-rump length (d) for the *TgTP6.4*$^{Tg/-}$ female × *TgTP6.4*$^{-/-}$ male cross. Each point represents a single fetus and fetuses are arranged by litters. Sexes were not distinguished and only litters with both tauGFP-positive and tauGFP-negative fetuses were included. Numbers of fetuses, means and *P*-values are shown. **(e-h)** Differences between within-litter means are shown for fetal mass and crown-rump length for tauGFP-positive and tauGFP-negative fetuses in each cross. *P*-values are shown for paired t-tests. Abbreviations: GFP-ve, tauGFP-negative; GFP+ve, tauGFP-positive.

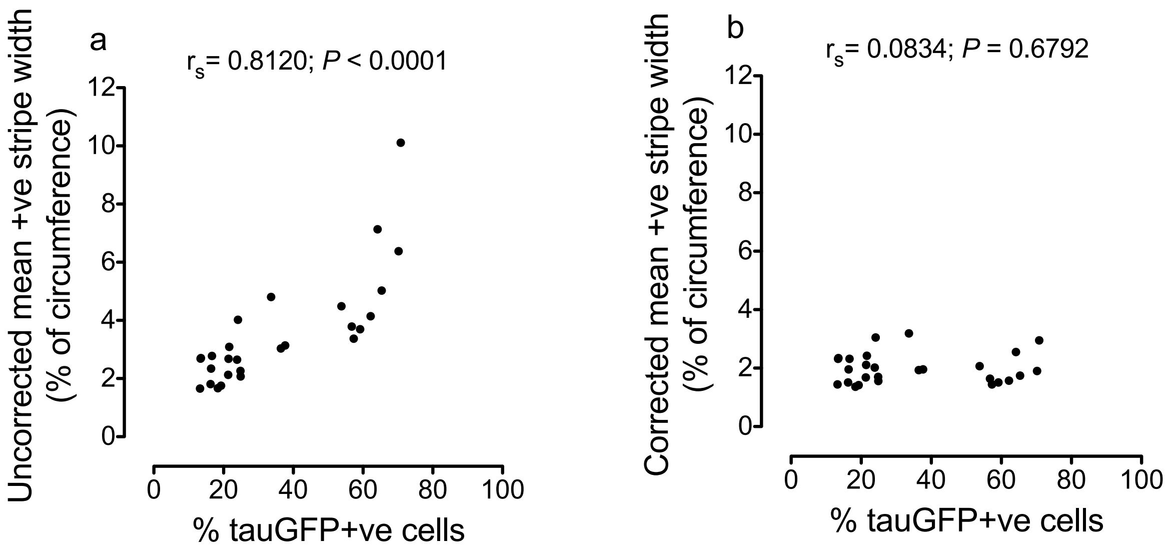

Figure S3. Relationship between mean stripe width and the percentage of tauGFP-positive cells in *TgTP6.4*$^{Tg/-}$ adrenal cortices. (a) The uncorrected mean tauGFP-positive stripe width for 27 *TgTP6.4*$^{Tg/-}$ adrenal glands varied widely. It was close to 2% of the adrenal circumference when the percentage of tauGFP-positive cells in the adrenal cortex was low but it was positively correlated with the percentage of tauGFP-positive cells. The Spearman correlation coefficient (r_s) is shown. This positive correlation is likely to be because stripes may comprise several adjacent coherent clones of cells and the average number of tauGFP-positive clones per tauGFP-positive stripe will increase with the percentage of tauGFP-positive cells in the adrenal cortex. **(b)** The corrected mean tauGFP-positive stripe width was not significantly correlated with the percentage of tauGFP-positive cells so allows comparisons among adrenals with different percentages of tauGFP-positive cells. The observed (uncorrected) mean tauGFP-positive stripe number was corrected by dividing it by the correction factor 1/(1-p), where p is the proportion of tauGFP-positive cells around the circumference as explained in the Methods.

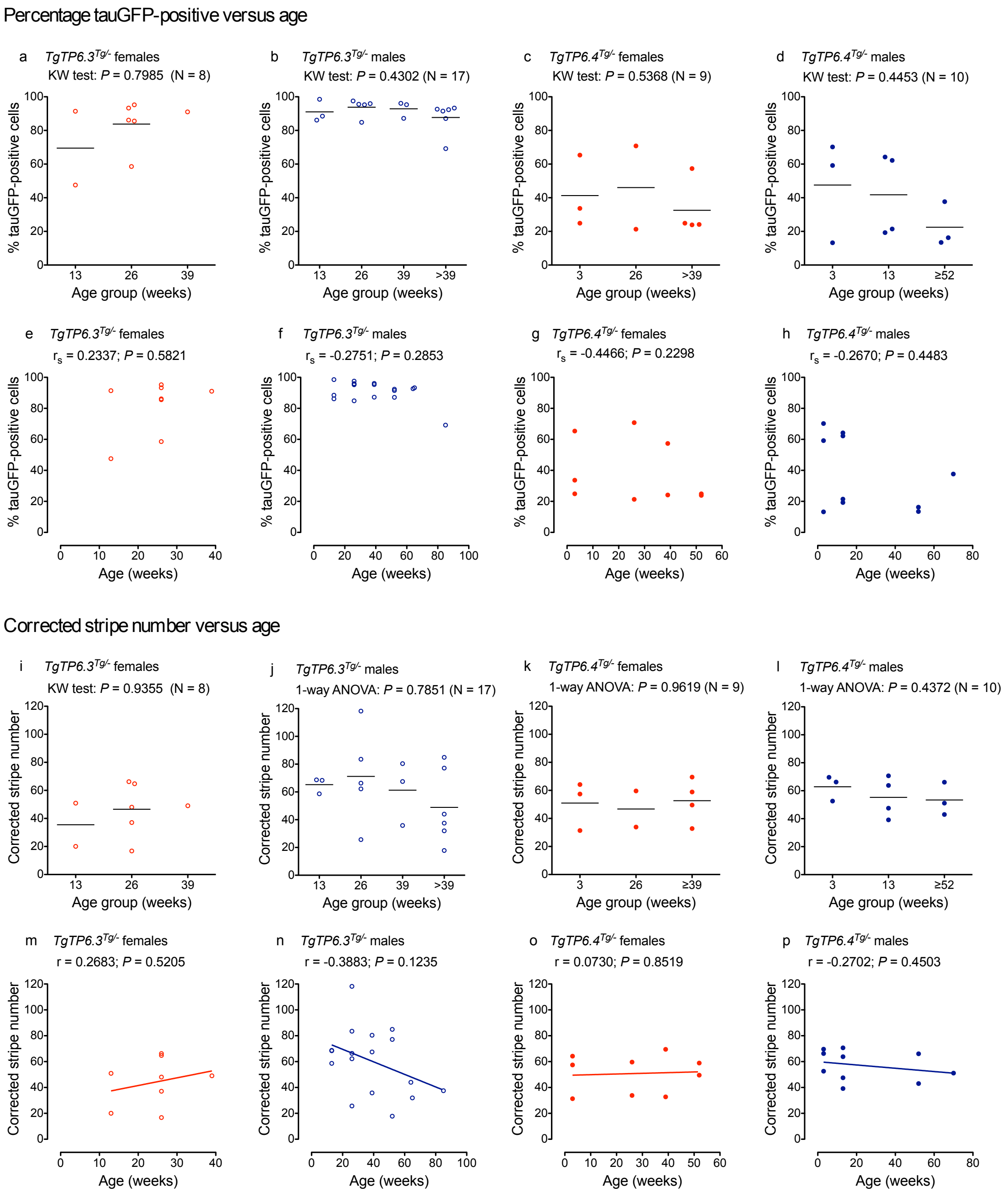

Figure S4. Age has no effect on percentage of tauGFP-positive cells or corrected stripe number in mosaic adrenal cortices of *TgTP6.3$^{Tg/-}$* and *TgTP6.4$^{Tg/-}$* mice. (a-d) There were no significant differences among age groups for the % tauGFP-positive cells in the adrenal cortex for (a) *TgTP6.3$^{Tg/-}$* females (b) *TgTP6.3$^{Tg/-}$* males (c) *TgTP6.4$^{Tg/-}$* females or (d) *TgTP6.4$^{Tg/-}$* males. Mice were grouped into three or more age groups and analysed by the Kruskal-Wallis test (KW test). **(e-h)** There were also no significant positive correlations between age and the % tauGFP-positive cells in the adrenal cortex for any of the four groups. Spearman correlation coefficients (r_S) are shown. **(i-l)** There were no significant differences among age groups for the corrected stripe number (tauGFP-positive stripes plus tauGFP-negative stripes) in the adrenal cortex for any sex and genotype combination. Mice were grouped into three or more age groups and analysed by the Kruskal-Wallis test (KW test) or 1-way ANOVA. **(m-p)** There were also no significant positive correlations between age and the corrected stripe number in the adrenal cortex for any sex and genotype combination. Pearson correlation coefficients (r) and the linear regression lines are shown but no lines differed significantly from horizontal. N = number of adrenal glands; one adrenal gland was analysed per mouse.

Table S1. FACS analysis of fetal brains

Sample	Percentage of cells in fluorescent region M1*	Ratio of M2 : M3 cells*	
		Percentage M2 (low fluorescence)	Percentage M3 (high fluorescence)
**GFP-positive *TgTP6.3*$^{Tg/-}$**			
Whole brain	95.26	13.37	86.63
Hippocampus	95.90	11.75	88.25
Cerebral cortex	98.52	11.69	88.31
Ventral telencephalon	96.29	24.17	75.83
Central and dorsal thalamus	92.99	8.72	91.28
Mid brain and hind brain	94.79	23.72	76.28
**GFP-negative *TgTP6.3*$^{-/-}$**			
Whole brain	0.55	98.18	1.82
**GFP-positive *TgTP6.4*$^{Tg/-}$**			
Whole brain	25.26	89.78	10.22
Hippocampus	52.84	87.12	12.88
Cerebral cortex	39.34	91.34	8.66
Ventral telencephalon	26.53	88.90	11.10
Central and dorsal thalamus	43.27	88.93	11.07
Mid brain and hind brain	40.23	88.05	11.95
**GFP-negative *TgTP6.4*$^{-/-}$**			
Whole brain	0.65	86.36	13.64

* Three gated regions were defined from a tauGFP-positive, *TgTP6.3*$^{Tg/-}$ sample. Region M1 included all fluorescent cells, M2 included cells with low fluorescence and M3 included cells with high fluorescence.
